# Patient Perspectives on Use of Video Telemedicine for Type 1 Diabetes Care in the United States during the COVID-19 Pandemic

**DOI:** 10.3390/endocrines2040040

**Published:** 2021-11-01

**Authors:** Stephanie S. Crossen, Crystal C. Romero, Lindsey A. Loomba, Nicole S. Glaser

**Affiliations:** 1 Department of Pediatrics, University of California, Davis, Sacramento, CA 95817, USA; 2 Center for Health and Technology, University of California, Davis, Sacramento, CA 95817, USA

**Keywords:** telemedicine, type 1 diabetes, patient-centered care, COVID-19 pandemic

## Abstract

The COVID-19 pandemic has resulted in widespread adoption of telemedicine for management of chronic conditions such as type 1 diabetes (T1D), but few data have been collected about the patient experience and perceived quality of care during this time. We surveyed members of the T1D Exchange patient registry and online community regarding their experiences with and opinions about telemedicine care during the pandemic. Among 2235 survey respondents, 65% had utilized telemedicine. The most common reasons for adopting telemedicine were providers not offering in-person care (66%), concerns about the health risks of in-person care (59%), providers offering (52%) or insurance covering (19%) telemedicine for the first time, and local or state orders to stay home (33%). Among telemedicine users, 62% felt video care was as effective as or more effective than in-person care, and 82% hoped to use telemedicine in the future. The most-cited reason for non-use of telemedicine was that providers were not offering it (49%). Our findings highlight the role of telemedicine in maintaining access to T1D care during the COVID-19 pandemic. Respondents’ satisfaction with telemedicine and interest in its continued use signifies the need for ongoing access to this care modality and for the development of telemedicine best practices within T1D care.

## 1. Introduction

The COVID-19 pandemic has resulted in the widespread adoption of telemedicine by medical practices across the United States [1] for the management of a wide variety of health conditions [2]. Type 1 diabetes (T1D) management is particularly well-suited to remote care due to its reliance on patient-generated health data and behavioral health interventions. Given these circumstances, the majority of U.S. endocrinologists [3] and the majority of diabetes clinics participating in the T1D Exchange Quality Improvement Collaborative [4] moved to entirely or predominantly “virtual” care in the spring of 2020. This transition was facilitated by policy changes under the U.S. Department of Health and Human Services, which provided parity in insurance coverage and reimbursement for telehealth—as compared to in-person office visits—during the pandemic [5].

In the wake of this dramatic transformation in care, it is essential to examine patient perspectives about how telemedicine has made T1D care more or less patient-centered during this time. Telemedicine has the potential to facilitate more patient-centered care through increased convenience, greater ownership of and engagement with one’s own diabetes data, and involvement of family members and/or medical providers who are physically distant. However, telemedicine can also add challenges in terms of new technology interfaces, communication barriers, and a decreased sense of personal connection with medical providers. Prior publications have demonstrated high patient satisfaction and time- and cost-savings with telemedicine interventions for T1D [6–11], as well as improved levels of engagement and self-efficacy in diabetes care [12,13]. In addition, telemedicine has been used successfully to improve glycemic control among individuals with T1D and individuals with type 2 diabetes [6,14–20]. This study aimed to better understand the experience of care via telemedicine for people with T1D, particularly in the context of the rapid and significant changes in healthcare delivery which took place during the COVID-19 pandemic.

We surveyed members of the T1D Exchange patient registry and T1D Exchange online community (formerly Glu) in the United States about their experiences with and opinions about the use of video encounters for T1D management during the COVID-19 pandemic. Our goals were to estimate how widespread the use of telemedicine has been for T1D management in the U.S. during this time and to learn more about the patient experience of T1D care when delivered remotely.

## 2. Materials and Methods

Survey questions were designed by the research team to elicit information about respondents’ experiences with and opinions about the use of video telemedicine for T1D-related medical care. Invitations to participate were distributed by T1D Exchange to its online community via email, Facebook, Twitter, and Instagram over an eight-week period, and to its online patient registry participants via email and an online portal over a four-week period in the fall of 2020. The T1D Exchange online community and T1D Exchange patient registry together include over 27,000 individuals who either have T1D or are caretakers (parents/guardians) for children with T1D and who participate voluntarily in these groups. Approximately half of these individuals are connected to T1D Exchange via email, and the remainder are connected primarily via social media.

Potential survey participants were asked to follow a QR code or hyperlink, which directed them to a website with additional information about the survey’s purpose, eligibility requirements, and expected time commitment for respondents (5–10 min). Eligibility requirements were an age of 18 years or older, living in the United States, and having T1D or being a caretaker for a child with T1D. An exclusion criterion was current incarceration, due to U.S. policies related to the inclusion of incarcerated individuals in scientific research. Individuals who met these criteria and chose to participate then followed an additional link and had to acknowledge their consent to participate and attest that they met eligibility requirements, at which point the survey was administered and respondent data were collected via a secure REDCap^™^ database.

The survey consisted of 13–18 multiple-choice questions, depending on participant responses. Only complete survey responses were included in the analysis. Univariate statistical comparisons of survey responses for sub-populations of interest were performed using the Pearson Chi-square test, with no expected—or actual—cell sizes containing fewer than five individuals. The UC Davis Institutional Review Board evaluated this study’s protocol and deemed it exempt from formal review. No personal identifiable information such as name, birth date, or contact information was collected from survey respondents.

## 3. Results

### 3.1. Survey Population

T1D Exchange sent email invitations for this survey to approximately 14,550 individuals. Social media impressions pertaining to this survey could not be precisely quantified but are estimated in the thousands. Overall, 2344 individuals initiated the survey and 2235 completed it; 109 incomplete survey responses were excluded from the analysis. Among our 2235 survey participants, 1974 (88%) responded as persons with diabetes (PWD) and the remaining 261 (12%) responded as caretakers of a child with T1D. Respondents were distributed across all fifty U.S. states and the District of Columbia, with the highest proportions of responses coming from California (10%) and New York (8%). Demographic and clinical characteristics of respondents are reported in Table 1.

### 3.2. Use of Video Care for T1D

**Figure 1.**
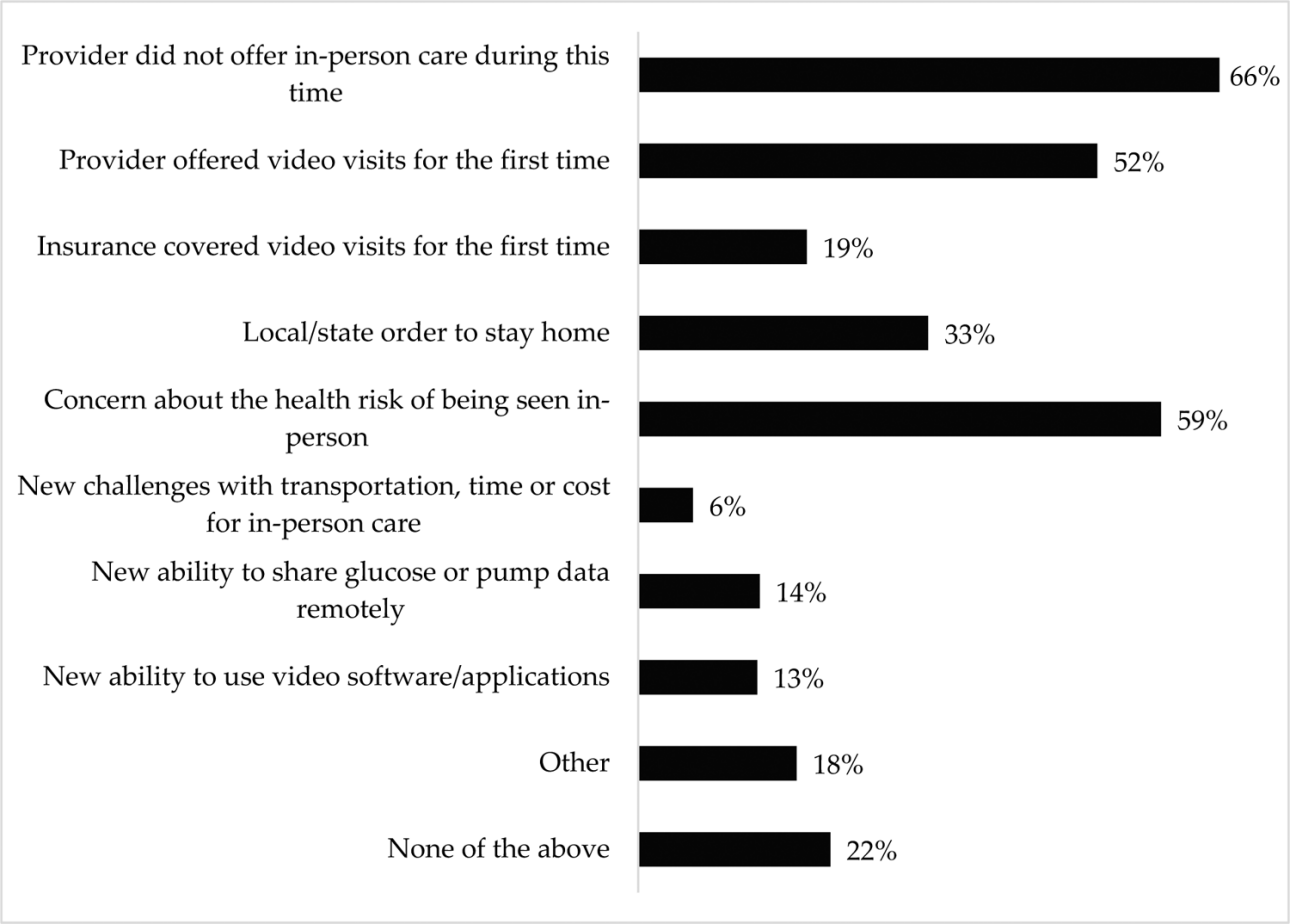
Factors Influencing Survey Respondents’ (*n* = 1393) Adoption of Video Care During COVID-19.

The majority of respondents (65% or 1452 individuals) had completed a diabetes medical appointment by video, and another 10% planned to use video for their next diabetes appointment; the remaining 25% had no plans to use video for their T1D medical care. Among respondents who had completed a video visit, 96% (1393 individuals) stated that their first video appointments took place during the COVID-19 pandemic, and they cited a variety of factors that led to their adoption of telemedicine during this time, as represented in Figure 1. The most-cited reason for using video care during COVID-19 was that providers were not offering in-person care. This was endorsed by 66% of respondents specifically and was mentioned by the majority of respondents who selected “other” and provided free-text comments.

Sub-group analysis revealed that respondents with public insurance were more likely than those with private insurance to report that video care adoption during COVID-19 was related to insurance covering video visits for the first time (27% vs. 16%, *p* < 0.001), while those with private insurance were more likely to report that their providers were not offering in-person care during the pandemic (68% vs. 57%, *p* < 0.001). Respondents of minority race or ethnicity and those from non-college-educated households were each less likely than their counterparts to report concern about the health risks of being seen in-person (47% vs. 60%, *p* = 0.005 and 53% vs. 61%, *p* = 0.010, respectively).

### 3.3. Opinions about Video Care for T1D

Among the 1452 respondents who had completed a video visit, 57% reported that they felt video care was as effective as in-person care for diabetes management, while 38% felt it was less effective and 5% felt it was more effective. In addition, large proportions reported that video care had saved them time (85%), stress (44%), and money (29%) as compared to in-person care. When asked if they would choose to continue using video appointments for diabetes care in the future, 17% responded that they preferred to receive diabetes care by video, 17% responded that they preferred diabetes care to be in-person, and 65% responded that they would choose to use video care for some appointments or in some circumstances.

Sub-group analysis demonstrated that respondents with hemoglobin A1c (HbA1c) levels ≥8% and those from non-college-educated households were each more likely than their counterparts to report that video care had not saved them time, money, or stress (17% vs. 10%, *p* = 0.003 and 18% vs. 9%, *p* < 0.001, respectively) and that they preferred all their diabetes care to be in-person (25% vs. 16%, *p* = 0.002 and 27% vs. 15%, *p* < 0.001, respectively). In addition, respondents from non-college-educated households were more likely to report feeling that video care was less effective than in-person care (46% vs. 36%, *p* = 0.001). However, for each of these sub-groups, the majority of respondents still felt that video care was as effective as or more effective than in-person care, that video care had benefitted them in some way, and that they would prefer to use video care for some appointments in the future.

Among the 777 respondents who had not yet completed a video appointment for T1D, 49% reported that the primary reason for this was that their providers had not offered video appointments, and respondents with public insurance were more likely than those with private insurance to report this limitation (56% vs. 47%, *p* = 0.029). The remaining 51% cited a variety of reasons, including preferring face-to-face care; feeling that video care would be of lower quality; or needing an HbA1c test, other laboratory tests, or a physical exam. Many respondents also reported that telemedicine felt unnecessary due to low perceived risk from COVID-19, already being in contact with their providers by phone or electronic messaging, or preferring to manage their T1D independently during this time. Sub-group analysis revealed that respondents with HbA1c ≥8% were more likely to report difficulty sharing glucose or pump data remotely (10% vs. 4%, *p* = 0.005) and concern about the quality of care by video (15% vs. 9%, *p* = 0.042). However, these reasons were rarely cited by that sub-population in comparison to the reason that their providers had not offered video care (50%).

## 4. Discussion

This survey of a large U.S. population living with T1D indicates that the use of video telemedicine encounters for T1D management has been widespread during the COVID-19 pandemic, and that video T1D care has been a new experience for most PWD (96%) who have utilized telemedicine during this time. Our study suggests that this shift in care was driven primarily by health insurance and medical providers changing the availability of telemedicine versus in-person appointments and secondarily by individual and community concerns about the health risks posed by in-person visits. Importantly, over a quarter of publicly insured respondents felt that changes in insurance coverage for telemedicine had been a key factor in their adoption of video care for T1D management. However, respondents with public insurance who had not utilized video care were more likely than their privately insured counterparts to cite providers not offering video care as the primary barrier. This confirms that access—or perception of access—to telemedicine has not been uniform during the pandemic, even among patients with similar insurance policies. It is also notable that over half of our respondents reported their providers were not offering any in-person T1D care at some point during the pandemic.

Our study suggests that among PWD who have used video care, the majority feel that it is as effective or more effective compared to in-person care (62%), that it allows them to save time in comparison to in-person visits (85%), and that they would want to continue using video telemedicine for T1D care in the future (82%). However, our sub-analyses suggest specifically that PWD from households with lower educational attainment and those with higher HbA1c levels are less likely to experience video care as convenient, effective, and desirable. In addition, many other respondents expressed feeling that video care was unnecessary because they were in contact with their providers by phone or electronic messaging or did not need provider input about T1D management during this time. These findings lead us to conclude that video telemedicine may be a patient-centered care modality for some individuals but not for all.

Two other published surveys about telemedicine use during the COVID-19 pandemic serve as interesting comparators for our results. In the spring of 2020, Horrell and colleagues surveyed over 2200 U.S. adults with a variety of non-diabetes chronic conditions who were engaged in online health communities, asking about their telemedicine use and satisfaction in the prior four months [21]. They found that 49% had completed telehealth visits and 39% of those who had engaged in telehealth felt that the virtual visit was as effective or more effective than in-person care. In addition, 27% of their cohort reported wanting to use telehealth for appointments after the COVID-19 pandemic. These numbers demonstrate substantial use and satisfaction with telehealth but are lower than the corresponding proportions of our own study population. This difference could relate to demographics (their population was similarly educated but older and more publicly insured), timing (their study took place in May 2020 and ours took place in August–October 2020, by which time the pandemic had progressed), and/or a difference in the utility of telemedicine for T1D versus other conditions.

To examine this latter possibility, we can compare our survey results to a global survey of youth and adults with T1D during the COVID-19 pandemic. Scott and colleagues surveyed over 7400 individuals with T1D in 89 countries (33% U.S. respondents) about telemedicine use and satisfaction [22]. Their study population overall had excellent glycemic control (mean HbA1c 7.1 +/− 1.2%) but a lower prevalence of insulin pump use (56%) than our cohort. Interestingly, only 8% of their respondents or approximately 600 individuals reported using telemedicine for T1D care, and another 20% reported using telephone visits. Of those using either video or phone care for T1D, 86% found these remote visits useful and 75% planned to use remote care in the future. Similar to our analysis, higher HbA1c was correlated with lower perceived utility of remote visits among remote care users. The much lower rate of video telemedicine use in this cohort may reflect the timing (March–May 2020) or telehealth availability globally compared to in the U.S., or it may indicate that they surveyed a fundamentally different subset of PWD than we captured in our study. However, the high rate of satisfaction among remote care users—with slightly lower satisfaction among PWD with elevated HbA1c—mirrors our own findings.

The strengths of our survey include a large study population and detailed information from respondents about the factors driving or discouraging telehealth adoption for T1D care from a patient standpoint. It adds importantly to the growing body of knowledge about how telemedicine is being used for T1D management across the U.S. and offers important insights about how much and in what ways this care modality may be patient-centered for PWD. A key limitation of our study is the fact that our survey population was predominantly White, non-Hispanic, college-educated and privately-insured, with excellent glycemic control and high use of therapeutic technology. Our results therefore reflect the use and perception of video T1D care among a very specific sub-population of PWD, and this limits their generalizability to other demographic groups. Multiple publications since the onset of the COVID-19 pandemic have demonstrated lower telemedicine use by populations with public insurance, minority race or ethnicity, and non-English language preferences [23–26]. These disparities in use may be partially driven by broadband internet and smartphone access and/or by differences in care providers and insurance coverage, but it remains unclear whether video-based telemedicine would be a patient-centered care modality for these populations if fundamental access barriers were overcome. We attempted to explore this question in our sub-analyses but acknowledge that even the subsets of our study population with minority race/ethnicity or from non-college-educated households likely differ in fundamental ways from the broader demographic groups to which they belong. Finally, our survey’s findings are limited to the use and perception of video telemedicine among individuals with T1D and therefore cannot be generalized to other types of remote care (e.g., telephone and electronic messaging encounters) or to type 2 diabetes management.

## 5. Conclusions

In conclusion, the majority of our 2235 survey respondents used video telemedicine for T1D care during the COVID-19 pandemic and want to continue video care in the future. These findings suggest that our healthcare system and diabetes community should work to maintain and improve access to telemedicine for all PWD after the pandemic. The differences in perceived access to and benefits from telemedicine among sub-populations of our survey respondents reaffirm the existence of disparities in telehealth use that have been noted by multiple other studies over the last year. In addition, the heterogeneity of opinions expressed by survey respondents about the utility of video care, and the concerns endorsed by many about the quality of care provided remotely, suggest that clinicians and researchers should focus on how to individualize not only the content but also the modality of T1D care so that it can be maximally effective for all PWD. Ongoing investigation to examine the experience of care is essential during rapid changes in healthcare delivery in order to ensure that patient-centered outcomes are considered alongside health outcomes and cost.

## Figures and Tables

**Table 1.** Demographic and Clinical Characteristics of Survey Respondents*.

Characteristics	Frequency (n)	Percentage (%)
**Total**	2235	100.0%
**Age (Years)**		
<13	123	5.5%
13–25	275	12.3%
26–50	920	41.2%
>50	913	40.9%
Not reported	4	0.2%
**Years Living with T1D**		
<5	235	10.5%
5–10	279	12.5%
11–20	423	18.9%
21–30	425	19.0%
>30	867	38.8%
Not reported	6	0.3%
**Devices Used for T1D**		
Fingerstick glucose meter	1322	59.1%
Continuous glucose monitor	1913	85.6%
Smart insulin pen	158	7.1%
Insulin pump	1441	64.5%
Hybrid closed-loop system	970	43.4%
**Most Recent HbA1c**		
<8%	1899	85.0%
8 to <10%	260	11.6%
≥10%	70	3.1%
Not reported	6	0.3%
**Gender**		
Female	1513	67.7%
Male	698	31.2%
Non-binary	15	0.7%
Other or Not reported	9	0.4%
**Ethnicity**		
Hispanic/Latino	83	3.7%
Non-Hispanic/Latino	2124	95.0%
Not reported	28	1.3%
**Race**		
American Indian/Alaska Native	15	0.7%
Asian	16	0.7%
Black or African American	40	1.8%
White	2081	93.1%
Other	78	3.5%
Not reported	5	0.2%
**Primary Insurance Type**		
Private	1615	72.3%
Public	564	25.2%
None/Self-pay or Other	45	2.0%
Not reported	11	0.5%
**Household Education Level**		
Did not graduate high school	33	1.5%
High school graduate	254	11.4%
Associate’s degree	238	10.6%
Bachelor’s degree	821	36.7%
Master’s degree	617	27.6%
Professional/doctoral degree	268	12.0%
Not reported	4	0.2%

T1D, type 1 diabetes; HbA1c, hemoglobin A1c.

* For caretaker respondents, responses pertain to the person with diabetes.
